# Management of Inflammatory Myofibroblastic Tumor of Urinary Bladder With Partial Cystectomy: A Case Report

**DOI:** 10.7759/cureus.111753

**Published:** 2026-06-29

**Authors:** Kieran T Raj, Amber Bauer, Sanjay Patel, Kelly Stratton

**Affiliations:** 1 Department of Urology, University of Oklahoma Health Sciences Center, Oklahoma City, USA; 2 Department of Urology, OU Health University of Oklahoma Medical Center, Oklahoma City, USA

**Keywords:** alk positive tumor, bladder neoplasm, bladder tumor, case report, hematuria, inflammatory myofibroblastic tumor, mesenchymal neoplasm, rare urologic tumor, urologic oncology

## Abstract

Inflammatory myofibroblastic tumors (IMTs) are rare mesenchymal neoplasms that can mimic malignant bladder tumors both clinically and radiographically. We report the case of a 46-year-old woman who presented with recurrent episodes of gross hematuria and clot retention. Imaging revealed a 6.2 cm bladder mass at the anterior dome without metastatic disease. She underwent transurethral resection, and pathology demonstrated a spindle cell neoplasm expressing anaplastic lymphoma kinase (ALK), desmin, and smooth muscle actin, while fluorescence in situ hybridization confirmed ALK gene rearrangement. Despite initial endoscopic management, persistent hematuria required a robotic-assisted partial cystectomy, which achieved complete resection with negative margins. The patient recovered well, and follow-up imaging and cystoscopy at six months demonstrated no recurrence.

This case highlights the diagnostic and management challenges of bladder IMTs, which often present with features that overlap with other malignancies of the bladder. While generally considered tumors of intermediate biologic potential, complete surgical excision remains the cornerstone of management. Bladder-sparing approaches, such as partial cystectomy, are effective in select patients and offer excellent outcomes when negative margins are achieved.

## Introduction

Inflammatory myofibroblastic tumors (IMTs) are a rare type of mesenchymal neoplasms. The incidence of these tumors is not well-established, though they are considered exceptionally rare. Histologically, IMTs are composed of mesenchymal spindle cells, accompanied by lymphoplasmacytic inflammatory infiltrate. Definitive diagnosis is established through histopathological examination, often after ruling out other malignant mesenchymal tumors. IMTs may arise in various body sites, with the abdominopelvic region being the most common location in adults and the thoracic/head and neck being more common in the pediatric population [1,2]. 

IMTs of the bladder are even more uncommon. Clinically, IMTs of the bladder often mimic urothelial carcinoma, presenting with hematuria, a bladder mass on imaging, irritative voiding symptoms, or anemia. The clinical and histologic overlap between IMTs and more common mesenchymal tumors, such as sarcomas, often complicates accurate diagnosis. Due to the rarity of this diagnosis, there is currently no consensus on optimal management. Here, we present a case of bladder IMT in a 46-year-old female, highlighting the diagnostic and management challenges posed by this rare entity.

## Case presentation

**Figure 1:**
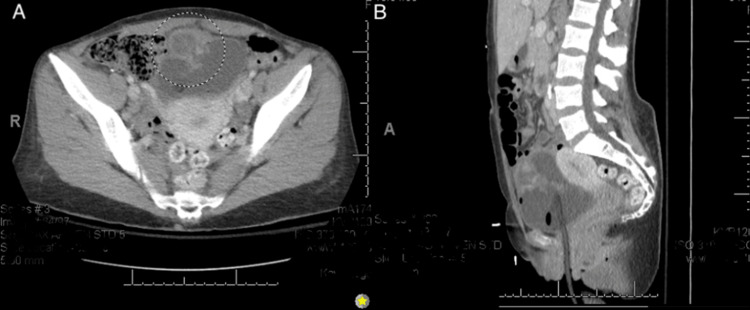
Contrast-enhanced CT of the pelvis (axial and sagittal views). (A) Axial CT image of the pelvis showing an anterior dome bladder mass (dotted circle) measuring approximately 6.2 cm. (B) Sagittal CT image of the pelvis confirming the intravesical location and extent of the bladder mass.

A 46-year-old female with a past medical history of ovarian cystectomy presented to the emergency department with three days of gross hematuria and clot retention. CT imaging revealed a 6.2 cm bladder mass at the anterior dome concerning for malignancy with no evidence of metastatic disease (Figure 1). She was also found to be anemic (hemoglobin 5.4 g/dL), requiring two units of blood transfusion. After failing conservative management with continuous bladder irrigation, the patient was taken for clot evacuation and transurethral resection of bladder tumor (TURBT). Cystoscopically, the solitary mass appeared nodular, with a white-and-tan fleshy appearance. The patient's urine subsequently cleared, and she was discharged with a catheter. Before her scheduled follow-up, the patient presented again to the emergency department with clot retention and anemia (hemoglobin 5.2 g/dL). She was transfused with one unit of blood and started on continuous bladder irrigation. Due to persistent hematuria requiring transfusions, the patient underwent repeat clot evacuation and fulguration. She was discharged two days after the procedure. 

**Figure 2:**
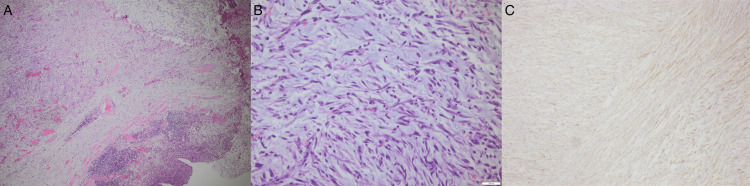
Histopathological findings. (A) Normal urothelium. (B) Spindle cell proliferation within a myxoid stroma. (C) Immunohistochemical staining demonstrating diffuse cytoplasmic positivity for anaplastic lymphoma kinase (ALK).

The pathology report showed a neoplasm composed of loosely arranged spindle cells in myxoid stroma with mixed inflammatory infiltrate and some tumor necrosis (Figure 2B). Immunohistochemical studies showed expression of anaplastic lymphoma kinase (ALK)-1, pankeratin, desmin, and smooth muscle actin (SMA) and were negative for DOG1, C-kit, and GATA3. Fluorescence in situ hybridization (FISH) for ALK was positive for an ALK gene rearrangement at 2p23 (Figure 2C). The pathology report concluded that the lesion was an ALK-positive inflammatory myofibroblastic tumor. 

On follow-up discussion with the patient about treatment options (close observation, repeat TURBT, or partial cystectomy), the patient elected for partial cystectomy. A partial cystectomy was performed using a robotic approach. Before docking, a cystoscopy was performed, and a resectoscope was used to delineate a 1 cm margin of healthy mucosa to aid in the discrimination of tumor boundaries. The robot was docked, and the bladder was mobilized with the anterior bladder mass identified. The bladder was then filled, and we made a cystotomy 3 cm away from the bladder mass and carried our incision to our premarked cauterized margin. The mass was circumferentially excised, and the bladder was closed using a barbed suture in two layers. The patient was discharged post-op day 1 with a catheter in place. CT cystogram three weeks later showed no leak, and the catheter was removed. Final pathology reconfirmed ALK-positive inflammatory myofibroblastic tumor with negative resection margins. The patient is undergoing surveillance with cystograms every three months and CTs every six months for one year. She remains without evidence of recurrence or urinary symptoms.

## Discussion

IMTs are exceptionally rare mesenchymal tumors, with an estimated incidence of fewer than one in one million people [3]. IMTs most commonly arise in the lung, mesentery, omentum, retroperitoneum, abdomen, and thorax, but can occur in nearly any soft tissue location, including the head, neck, and extremities. They primarily affect children and young adults, although cases across a broad age range have been documented [2-6]. Primary bladder IMTs are even less common, accounting for less than 1% of all bladder tumors [4]. When arising in the bladder, IMTs often present with symptoms that overlap with those of bladder malignancies, including gross hematuria, irritative voiding symptoms, and, occasionally, clot retention.

Grossly and microscopically, IMTs are typically well-circumscribed, solitary or multinodular masses that are composed of spindle cells in a myxoid stroma, often with inflammatory infiltrates of lymphocytes, plasma cells, or eosinophils [5]. Variants include myxoid or vascular patterns, compact spindle cell patterns, and fibrous hypocellular patterns [6,7]. The most common pattern observed in bladder IMTs is the spindle cell pattern [4]. Radiographically, these lesions appear similar to urothelial carcinomas or other malignant bladder masses, further complicating diagnosis [4,6,8]. Therefore, a high index of suspicion and thorough histopathological examination are essential for accurate diagnosis. 

Immunohistochemically, IMTs typically express vimentin, SMA, and desmin, with variable expression of ALK. ALK positivity is observed in approximately 50%-60% of cases [5], and some studies suggest that ALK-positive IMTs may have a better prognosis and response to targeted therapy, such as ALK inhibitors [9-12]. Conversely, ALK-negative tumors are more frequently associated with locally aggressive behavior and a higher risk of metastasis [4,13]. However, this association is debated, as other studies have found no statistically significant correlation between ALK reactivity and clinical outcomes [1]. 

Differentiating IMTs from other malignant tumors, such as sarcomas and carcinomas, can be challenging. Sarcomas typically display more aggressive histological features, including cellular atypia and high mitotic activity. Carcinomas, particularly urothelial carcinomas, may overlap in presentation, but the presence of myofibroblasts and lack of epithelial components are clues used to differentiate the two. Immunohistochemical staining is critical for distinguishing IMTs from morphologically similar tumors such as sarcomas and urothelial carcinomas. IMTs can typically be distinguished by their expression of SMA, desmin, and vimentin. While ALK-1 is positive in many cases, its presence varies and is not exclusive to IMTs [5]. In contrast, sarcomas may overlap in SMA and desmin positivity but are characteristically ALK-negative and display more aggressive histologic features, including high mitotic activity, nuclear pleomorphism, and tumor necrosis. Urothelial carcinomas can be readily distinguished by their expression of GATA3, p63, and cytokeratins 7 and 20, none of which are typical of IMTs [14]. These immunohistochemical patterns, when interpreted alongside histologic features such as myxoid stroma and inflammatory infiltrates, are essential for accurate diagnosis and exclusion of malignant mimics. 

**Figure 3:**
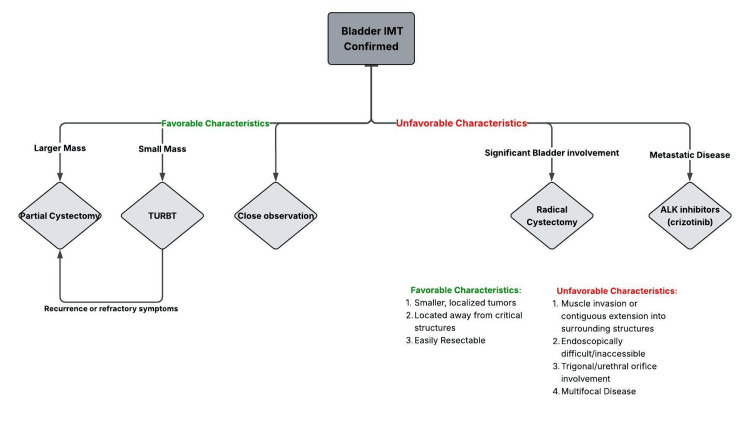
Proposed treatment algorithm. Image created by Kieran T. Raj, Amber Bauer, and Sanjay Patel using Adobe (Adobe Inc., San Jose, CA). TURBT, transurethral resection of bladder tumor; ALK, anaplastic lymphoma kinase

Given the generally indolent nature of IMTs, treatment is primarily focused on alleviating symptoms and achieving complete tumor extirpation. Bladder-sparing techniques, including TURBT and partial cystectomy, are the preferred approaches for managing bladder IMTs [4,6,15]. Ideal candidates for bladder-sparing procedures include those with smaller tumors, favorable tumor location, and when the surgeon's comfort level allows for complete resection. Positive surgical margins are associated with a higher risk of recurrence; therefore, complete excision is critical for optimal outcomes [13]. Radical cystectomy (RC) is generally reserved for cases involving very large tumors, those located in difficult or inaccessible areas, or when there is a misdiagnosis of a more aggressive malignancy. The decision to proceed with RC is based on the feasibility of bladder-sparing techniques and overall tumor characteristics. Based on these principles, we propose a treatment algorithm that highlights favorable versus unfavorable characteristics and outlines corresponding management strategies (Figure 3).

The role of ALK expression in guiding treatment is an area of ongoing research. ALK-positive IMTs are generally considered to have a more favorable prognosis and may respond better to targeted therapies, such as ALK inhibitors. One such ALK inhibitor, crizotinib, has demonstrated promising results in ALK-rearranged IMTs and has been proposed as a potential neoadjuvant or salvage therapy for unresectable or refractory cases [7]. However, due to its ill-characterized toxicity profile and limited data on efficacy in bladder IMTs specifically, its use is generally considered a last-resort option. Surveillance following surgical resection typically involves periodic cystoscope examinations and imaging studies to monitor for recurrence. The optimal follow-up interval is not well-defined, but surveillance every three months with cystoscopy and every six months with imaging for the first year is a commonly adopted approach [4,6,15-17].

The prognosis for bladder IMTs is generally favorable, owing to their typically benign behavior, low recurrence rates, and rare incidence of metastasis. This favorable outlook is further reflected in published outcomes, where recurrence rates following bladder-sparing surgical approaches remain low across multiple studies. In a series of 14 patients, Chen et al. reported that 71.4% underwent partial cystectomy and 21.4% underwent TURBT, with only one case of local recurrence (7.1%), which was managed successfully with repeat TURBT [15]. Similarly, Teoh et al. reviewed 49 patients and found a recurrence rate of 10.2% and a five-year disease-specific survival rate of 99% [6]. Hage et al., in their systematic review of 182 cases, described favorable outcomes with bladder-sparing techniques and a recurrence rate of 4.3%, particularly in cases with incomplete resection [4]. Although long-term data on aggressive or metastatic IMTs remains limited, rare instances of distant spread have been documented. Several studies report a metastatic rate of less than 5%, with the lungs, liver, and brain being the most commonly affected sites [1,9,18]. Despite these rare events, overall survival remains favorable, with reported five-year survival rates ranging from 77% to over 90%, depending on tumor location and completeness of resection [11,19]. Bladder-sparing procedures, including partial cystectomy and TURBT, are associated with excellent outcomes when complete excision is achieved. In contrast, RC is typically reserved for tumors that are unresectable, misdiagnosed as malignant, or recur despite conservative management. However, long-term prognosis in patients requiring RC or those with metastatic disease remains poorly characterized due to the rarity of such cases. Continuous follow-up and surveillance remain essential, particularly for patients with positive surgical margins, given the risk of local recurrence. 

## Conclusions

IMTs of the bladder are rare neoplasms that generally carry a favorable prognosis with a low risk of metastasis. The favorable prognosis is attributed to the typically benign nature of these lesions, though recurrence and local invasion can occur. In this case, a 46-year-old female presented with gross hematuria and clot retention, with imaging revealing a 6 cm mass at the anterior bladder dome. She underwent a partial cystectomy due to recurrent bleeding and clot retention. Postoperatively, she remains on surveillance without evidence of recurrence on follow-up cystoscopy and imaging.
